# Multimode entanglement in reconfigurable graph states using optical frequency combs

**DOI:** 10.1038/ncomms15645

**Published:** 2017-06-06

**Authors:** Y. Cai, J. Roslund, G. Ferrini, F. Arzani, X. Xu, C. Fabre, N. Treps

**Affiliations:** 1 Laboratoire Kastler Brossel, UPMC-Sorbonne Universités, CNRS, ENS-PSL Research University, College de France, CC74, 4 Place Jussieu, 75252 Paris, France; 2 State Key Laboratory of Precision Spectroscopy and Department of Physics, East China Normal University, Shanghai 200062, China; 3 Laboratoire Matériaux et Phénomènes Quantiques, Sorbonne Paris Cité, University of Paris Diderot, CNRS UMR 7162, 75013 Paris, France

## Abstract

Multimode entanglement is an essential resource for quantum information processing and quantum metrology. However, multimode entangled states are generally constructed by targeting a specific graph configuration. This yields to a fixed experimental setup that therefore exhibits reduced versatility and scalability. Here we demonstrate an optical on-demand, reconfigurable multimode entangled state, using an intrinsically multimode quantum resource and a homodyne detection apparatus. Without altering either the initial squeezing source or experimental architecture, we realize the construction of thirteen cluster states of various sizes and connectivities as well as the implementation of a secret sharing protocol. In particular, this system enables the interrogation of quantum correlations and fluctuations for any multimode Gaussian state. This initiates an avenue for implementing on-demand quantum information processing by only adapting the measurement process and not the experimental layout.

## Abstract (web summary)

Multimode entanglement is an important resource for quantum information processing, but setups are often able to generate specific configurations only. Here the authors present an on-demand reconfigurable multimode entangled state source, realizing thirteen cluster states of various sizes and connectivities.

Inseparability, that is, the impossibility of treating as separable entities physical systems that have been generated in an entangled, non-factorable quantum state, even though the systems are no longer coupled to each other by a physical interaction, is one of the most puzzling properties of the quantum world^1^,^2^. The consequences of this quantum property have been harnessed in a range of applications, including quantum teleportation^3^,^4^ and quantum computation^5^,^6^. To compete with classical computers, quantum computers need to employ a large number of quantum systems that are created in appropriately designed entangled states, on which quantum processing operations can be performed before the quantum state is subject to decoherence. This multipartite quantum system is often termed a ‘quantum network', and the individual quantum-correlated systems comprise the network ‘nodes'. The generation and use of large quantum networks raise numerous experimental and theoretical issues that are the subject of intense research. For instance, significant effort has recently been directed towards defining specialized metrics that assess the presence of multipartite entanglement^7^,^8^,^9^,^10^ as well as characterize the ‘quality' of such a quantum resource in view of quantum computing applications. This issue is still the subject of debate throughout the community.

The majority of hitherto studied systems have employed qubits (that is, material-based two-level systems, such as ions or quantum dots) as the nodes of a quantum network. In this case, the parties comprising the multipartite quantum network are well-defined physical objects, and multipartite entanglement amongst nodes appears as a many-body property where each party is physically separated from the others and can be measured independently^11^. Furthermore, while a multitude of experiments have demonstrated the construction of multimode entanglement, the experimental architecture typically realizes one specific structure and is not reconfigurable^12^,^13^,^14^. Hence, a general study on the diversity of networks that are attainable from a single fixed resource has not been performed.

For that purpose multimode optical sources are ideal candidates. Indeed, multimode entanglement properties are governed by the initial quantum state and by the measurement process. More specifically, multipartite entanglement is not anymore merely an intrinsic property of the source, but also the result of a complex interplay amongst the source, act of measurement, and possibly post-processing that acts on the measurement results^15^,^16^,^17^. One should stress that this setting is not fully equivalent to a quantum network, as in its general acception this concept requires distant physical nodes on which quantum information is processed. However, within the measurement based framework, quantum information can still be processed with purely optical systems, and thus the difference in application between these two different types of network becomes tiny. To avoid confusion, we decided to name these systems all-optical quantum graph.

In the present work, we tailor the measurement bases of a multimode optical quantum source by shaping the local oscillator of the homodyne measurement. This enables accessing a multiplicity of quantum correlations structures without any modification of the experimental arrangement. As a result, a direct study is accomplished of the scalability and versatility of graph connectivities that may be forged from a single resource. This new avenue paves the way for configurable, adaptive, and scalable quantum information processing whose possibilities are still largely unexplored, both theoretically and experimentally.

After explaining how measurement-based all-optical quantum graphs can be implemented, we introduce the experimental platform, which is based on parametrically generated ultrafast frequency combs whose temporal/spectral structure is exploited to carry multimode quantum information^18^. The use of ultrafast pulse shaping combined with homodyne-based projective measurements allows the on-demand construction of various multimode quantum correlation structures. As a practical illustration, we focus in particular on the generation of cluster states that are fabricated from the same light resource. Subsequently, a multipartite quantum secret sharing proposal is implemented by making use of one of the generated cluster states.

## Results

### Preliminary considerations

We consider the electric field quantum operator 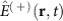 (a scalar field is assumed for simplicity), which is written in a general form as:


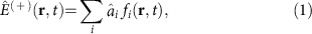


where *f*_*i*_(**r**,*t*) constitute a basis of optical modes (that is, orthonormal solutions of Maxwell's equations with specific boundary conditions), 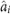 are photon annihilation operators in the mode of spatio-temporal shape *f*_*i*_(**r**,*t*), and an overall multiplicative factor has been ignored for simplicity. This set of modes can be placed in a multimode entangled state, whose correlations structure can be described as a graph state^19^.

Compared to ‘material-based quantum networks', photonic networks exhibit unique properties that include a relative insensitivity to decoherence but also an ability to arbitrarily change the mode basis. Towards this end, the field 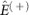 may be rewritten as:


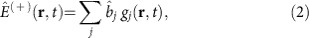


in which {*g*_*j*_(**r**, *t*)} represents another mode basis while 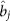 are the associated photon annihilation operators in the mode *g*_*j*_. A transformation from the original modal basis and annihilation operators to another is accomplished by means of a unitary transformation:


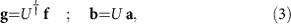


where *U* is a unitary transformation acting on the vector space of modes, and the vectors **g**, **f**, **b**, **a** have respective components *f*_*i*_, *g*_*j*_, 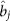, 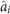. The potential for examining a given quantum state in an arbitrary modal basis is one of the most important features of multimode quantum optics, whose equivalent has not been demonstrated for material qubits so far. Importantly, it is possible to experimentally access the properties of a given mode (for example, *f*_*i*_) using balanced homodyne detection in which a local oscillator is temporally and spatially sculpted in the same mode^20^. Such a measurement also has the potential to arbitrarily reconfigure the projection operator that acts on the multimode optical state of interest^15^,^21^,^22^ in a spirit closely related to measurement based quantum computing^6^,^23^,^24^.

### Measurement based all-optical quantum-graphs

The Bloch Messiah decomposition^25^ states that any pure multimode Gaussian quantum state of light can be reduced to a set of uncorrelated squeezed vacuum states in an appropriately chosen mode basis of annihilation operators **a**^psqz^ (the array of modes are conventionally taken to all be squeezed in the *p*-quadrature of the field). This implies that the modes of any Gaussian all-optical quantum graph may be constructed from a set of squeezed modes by implementing a proper change of mode basis. In practice, a graph of interest may be fashioned by applying a unitary transformation *U*_net_ to a set of independently squeezed modes^24^, which allows for the annihilation operators **b**^net^ of the graph to be described as


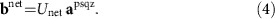


The unitary transformation *U*_net_ is conventionally implemented by means of a suitable arrangement of linear optical elements, including beamsplitters and phase shifters, and several pioneering experiments have demonstrated this approach^14^,^26^,^27^. As *U*_net_ mathematically corresponds to a general basis rotation, an alternative, but equivalent, manner in which to reveal the optical graph is to measure the multimode beam in the appropriate basis. Such a basis change can be implemented with a mode-selective detection system, which is the novel approach that is considered in this work.

Considering that any basis change is at hand, the realization of an arbitrary Gaussian all-optical quantum graph may start from any highly multimode non-classical state. In the present situation, the parametric down conversion of an optical frequency comb generates full multimode entanglement in the frequency basis^28^. The spectral domain in which the downconversion occurs is described with a set of ‘frequency-pixel modes' **h**^pix^ (in practice, they do not correspond to single frequency components but instead to a given frequency band matching the spectral resolution of the detection system) with corresponding annihilation operators **a**^pix^. These optical modes constitute an approximate basis on which the squeezed modes **f**^psqz^ can be decomposed. The set of annihilation operators corresponding to the squeezed modes may then be written as **a**^psqz^=*U*_sqz_**a**^pix^, where *U*_sqz_ is the corresponding unitary transformation whose phase degrees of freedom are chosen such that **a**^psqz^ are squeezed along the *p* quadrature. After applying the unitary transformation *U*_net_ corresponding to the graph of interest, the transformation becomes


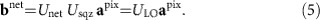


**Figure 1:**
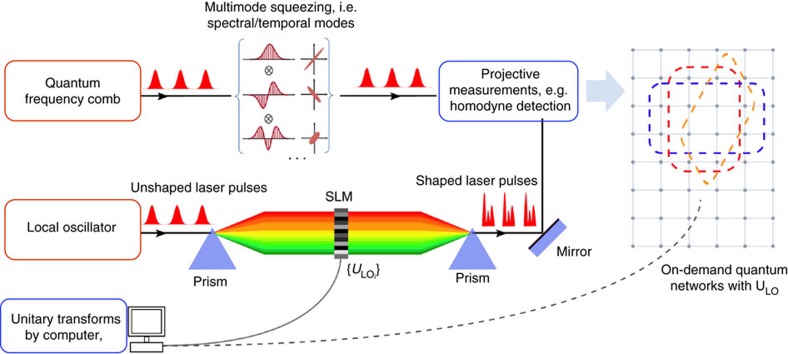
Experimental setup for the all-optical quantum graph. The system is based on a quantum frequency comb^30^ and homodyne detection with a customized local oscillator. The quantum frequency comb is a multimode squeezed state, in which each squeezed mode possesses a specific spectral structure. Consequently, quantum correlations exist in the frequency-band basis^18^. On-demand all-optical quantum-graph correlations within the frequency comb are revealed via projective measurements, which consists of homodyne detection in a suitable basis. The local oscillator (LO) is sculpted into the appropriate pulse shape by a computer-programmed spatial light modulator (SLM). The subsequent measurement of the quantum state with this shaped LO realizes the desired graph unitary transformation *U*_LO_. The top inset represents the spectral eigenmodes with corresponding squeezing ellipse in phase-space representation. The grid graph in the right suggests that via measurement one can access on-demand multimode entanglement with specific connectivities.

Consequently, every all-optical quantum graph possesses a unitary matrix *U*_LO_ that allows it to be related to the frequency-pixel mode basis. As seen in Fig. 1, this transformation can be implemented by a series of homodyne measurements with the Local Oscillator (LO) in the appropriate spectral shape.

### The quantum resource

The multimode quantum resource is formed from the parametric downconversion of an ultrafast pulse train. A 76 MHz pulse train delivering ∼120 fs pulses centered at 795 nm is frequency doubled, which serves to pump a *χ*^2^ non-linear crystal in a low finesse cavity. This pump source is composed of about ∼10^5^ single frequencies, each of which can be the potential source of ∼10^5^ different pairs of down-converted photons^29^. The resultant downconverted source can be characterized by either directly assessing its entangled character in the frequency domain or by extracting the eigenmodes of the downconversion process^18^,^28^,^30^. Given the highly multimode character of the downconverted comb, the limits of the quantum resource are determined by the quality of the detection process^31^.

Detection is performed with pulse shaped homodyne detection (Fig. 1). To reach a highly multimode regime, a high resolution pulse shaper is used along with high quantum efficiency detectors (see Methods). The resolution of the pulse shaper is ∼0.06 nm per pixel in a 30 nm band centered at 795 nm. The LO field, which originates from the same source laser, undergoes both amplitude and phase spectral shaping with this device, and the resulting shape defines the detection mode of the homodyne setup^32^,^33^.

**Figure 2:**
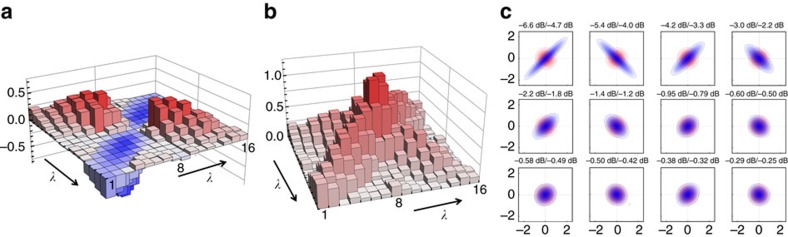
Multimode quantum resource. (**a**,**b**) 16-partite covariance matrix in the frequency-pixel basis in phase and amplitude quadrature, rexpectively. This correlation matrix is obtained with balanced homodyne detection where the spectrum of the local oscillator is divided into 16 frequency bands of equivalent width. The shot noise contribution has been subtracted from the diagonal for increased visibility, and axis values are normalized to vacuum noise. (**c**) Inferred squeezing of the eigenmodes (corrected/not corrected from measurement losses). The ellipses (blue) represent the squeezing of the twelve leading modes. The circles (red) represent vacuum fluctuations for comparison. Twelve of the sixteen modes are squeezed. The measurement results in panels **a**–**c** have been corrected for electrical dark noise and a 15% optical loss in the measurement processes.

To characterize the initial quantum resource, the LO spectrum is first divided into 16 frequency bands of equal bandwidth (∼0.8 nm). These bands correspond to the pixel modes of equation (5). With the same general strategy as the one presented in ref. 18 except for a direct computer acquisition of the noise data (see Methods section), the accurate measurement of large covariance matrices is accomplished in a short time period (around 1 s). The resultant amplitude and phase covariance matrices are shown in Fig. 2a,b. It is important to stress that, as was demonstrated in previous publications^18^, our system does not exhibit any measurable amplitude-phase correlation, as expected from parametric down conversion from a constant phase pump pulse. By applying a Bloch-Messiah decomposition to these matrices^25^, 16 eigenvectors and eigenvalues are extracted, which correspond to the orthogonal squeezed spectral modes and their respective squeezing values (Fig. 2c). These modes comprise the input basis of our all-optical quantum graph, which consists of 12 significantly squeezed modes with squeezing values ranging from −0.3 to −6.6 dB. To better assess the properties and quality of our system, the squeezing values presented in Fig. 2b are corrected for the homodyne detection visibility and detection losses (15% in total, including the visibility). They correspond to the available ressource independent from the measurement system. Henceforth, only the dark noise contribution to the data (very low in our case, from −10 to −15 dB depending on the actual power impinging the detectors) is removed for the graphs presented in the remainder of this work, and no correction is applied for detection losses.

To summarize, the process of parametric downconversion provides the link between the 16-mode operators in the measurement basis 
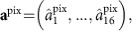
 and those in the squeezed basis 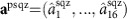. This link is the experimentally measured unitary transformation *U*_sqz_, which acts as 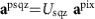. To reveal any all-optical quantum graph, the local oscillator is shaped according to equation (5). The high resolution of the pulse shaper allows for a fine reconstruction of the graph, at the expense of detecting only one mode at a time. It allows for accessing any of the modes or witness inequalities (see next section) that characterize a given quantum graph, but with the restriction that they cannot be revealed simultaneously.

### Continuous variable cluster states

With an eye towards applications in measurement based quantum computing^6^,^23^,^24^, we first reveal a series of different continuous variable (CV) cluster states. CV Cluster states are multimode Gaussian states for which specific quadrature combinations, called nullifiers, are defined by


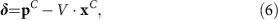


and should satisfy the relation Δ^2^***δ***→0 in the limit that input squeezing tend to infinity^23^,^24^. In this formulation, **x**^*C*^ and **p**^*C*^ are, respectively, the amplitude and phase quadratures of the cluster nodes **a**^*C*^=**x**^*C*^+*i* **p**^*C*^, and *V* is the adjacency matrix of the graph and defines the connectivity of the cluster state. In this work we exclusively consider weight +1 cluster states.

**Figure 3:**
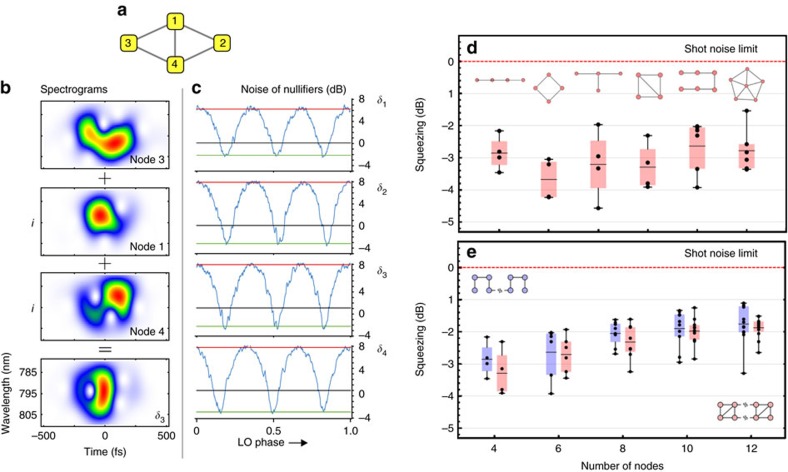
Simulation of CV cluster states. (**a**,**b**,**c**) Witness measurement. (**a**) Graph of the diagonal-square four-node cluster state as defined by the adjacency matrix *V*. (**b**) A sample of the spectrograms that correspond to this cluster state. The leading three spectrograms depict the pulse shapes corresponding to the optical nodes indicated in the corner of each image, and the final spectrogram represents the nullifier *δ*_3_ for node 3 as defined by equation (6), which is formed as the spectral superposition 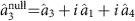. (**c**): To measure the nullifier variances associated with the correspondingly generated cluster state, the pulse shaper sculpts the LO in the spectral form associated with each of the four nullifiers, and the resulting four variance curves as a function of the global LO phase are shown. Each of the four nullifiers exhibits noise statistics below the shot noise limit (black lines). (**d**,**e**) Versatility and scalability. Nullifier squeezing values of various cluster states possessing between 4 and 12 nodes are presented as box plots. The black points are the individual nullifier variances, the pink rectangles depict the first and third quartiles of the data, the black line contained in the rectangle is the nullifier mean, and the black whiskers indicate the upper and lower extrema of the nullifier collection. The red dashed lines in (**d**,**e**) represent the shot noise limit. All of the nullifier variances are below the shot noise limit, which is a signature of the presence of the targeted cluster states. In (**e**), the variances of the n-mode linear (left, blue) and diagonal square (right, pink) cluster states are compared. The noise variances in (**d**,**e**) are only corrected for electrical dark noise.

A unitary transformation *U*_*C*_ may be used to represent each cluster node as a complex superposition of the uncorrelated squeezed states embedded within the comb output. The individual nullifier relations as defined by equation (6) also correspond to specific superpositions of the squeezers. Consequently, a particular spectral mode may be associated with each of these nullifiers. As an example, the pulse shapes that characterize each node of a diagonal-square four-node cluster state are shown in Fig. 3a. The optical mode corresponding to the third nullifier *δ*_3_ is constructed by shaping the LO into a form that corresponds to the summation of the amplitude quadrature of cluster node three with the phase quadratures of cluster nodes one and four (as specified by equation (6)), that is, 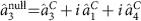. This shaped LO pulse form is projected onto the multimode entangled state by homodyne detection, which allows for measuring the nullifier variance of the associated cluster node. The nullifier variances for the other modes are obtained in a similar fashion. As seen in Fig. 3c, all of the nullifiers variances possess squeezing values between −2 and −4 dB, which indicates the presence of quantum correlations with CV cluster state structure.

Note that when a cluster state is generated by means of a linear optics transformation Uc, if the input squeezing is finite then the generated cluster departs from the target one. In particular, its weights may become complex valued^19^. Yet, if the squeezing level is high enough, the variances of the nullifier corresponding to the target adjacency matrix *V* may still be below the shot noise for the linear-optics generated cluster^27^. This is indeed what we assess with our method. We test nullifiers corresponding to the real-weighted adjacency matrix *V* and find that their variances lie below the shot noise, despite having employed the linear-optics construction method.

This scheme was also exploited to fabricate additional cluster states with nodes that range in number from 4–12. In Fig. 3d, the nullifiers corresponding to a number of 4- and 6-node cluster graphs are represented along with the corresponding connectivity structure. These variances are once again measured by a suitable programming of the pulse shaper as prescribed by equation (6), in which the adjacency matrix *V* for each cluster is given by the geometrical figure above the corresponding nullifier. Additionally, the scalability with respect to cluster dimensionality is analysed in Fig. 2b (b), where linear and diagonally-connected square clusters are constructed from a number of nodes that ranges from 4–12. Both of these structures possess a set of nullifiers that lie below the shot noise limit for all considered dimensionalities, which is a signature of the presence of these various graph states.

Importantly, the unitary transformation *U*_*C*_ leading to a given cluster state is not unique. For the situation in which each of the input squeezers possesses the same degree of squeezing, a basis rotation on these modes before the *U*_*C*_ transformation would not change the obtained graph connectivity. However, in the case of disparate input squeezing levels, the measured nullifier variances depend on the specific choice of the unitarity transformation. The present work optimizes the choice of the matrix *U*_*C*_ among all of the possible basis rotations that yield a given cluster state with a specific graph structure^34^. This is accomplished with an offline optimization that minimizes the mean of the cluster nullifier variances for a specific structure given the experimental input squeezers of Fig. 2. As a result, the mean of the nullifier variances is approximately equal across the examined cluster series as seen in Fig. 3d,e, which indicates that the finite resources available have been optimally allocated (see also the Method section). Together with inseparability criteria assessed below, the nullifiers values are a witness for successful cluster generation. Among the variations that persist following optimization, it is observed that cluster states with a higher connectivity exhibit a lower mean nullifier variance for a fixed number of modes^35^.

**Figure 4:**
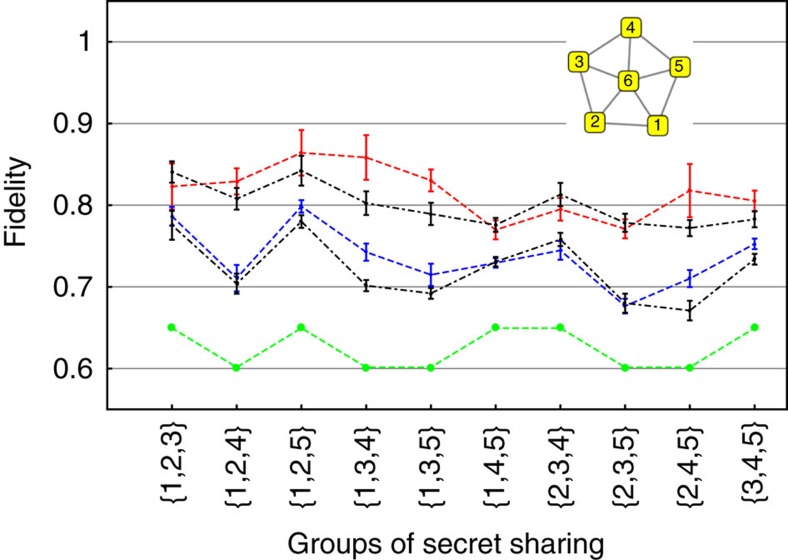
Experimental fidelities for the simulation of quantum secret sharing. The graph state that is used in the secret sharing protocol is shown as an inset in the upper right portion of the figure. Nodes 1–5 constitute the players and node 6 is the dealer. The horizontal axis of the plot shows all 10 of the possible combinations for a three member access party. The red and blue dashed lines are the measured fidelities of the reconstructed secret given a −6.6 and −4.5 dB squeezing resource, respectively. The black dashed-point curves are inferred from the individual squeezing of the eigenmodes through the use of equation (9). The green dashed curve corresponds to the fidelity with ordinary vacuum replacing the squeezed resource. All of these fidelities are directly measured and only contain a correction for the electrical dark noise. The error bar in the all curves represents uncertainty in fidelity reconstruction, calculated with the same methodology as cluster nullifiers uncertainty.

For genuine demonstration of cluster states, it is usually understood that beyond the nullifiers, one has to assess the inseparability of the multimode state. Generally speaking, our state has been proven to be fully entangled in ref. 36. However, it is interesting to assess inseparability for the actual nodes of a given cluster. To do so, because in our system it is possible to obtain the full covariance matrix, we have been using the PPT (positive partial transpose) criteria^37^. For any partition of any of the clusters demonstrated in this article, we find inseparability. More specifically, as an example we focus here on the six mode graph with the structure shown in Fig. 4 as it will be the one relevant for secret sharing. We find that for any bipartition, the smallest eigenvalue of the partially transpose matrix is comprised between −0.20 and −0.5 (in shot noise units), with a mean of about −0.40. The most inseparable partition being the one between nodes {1, 2, 3, 4} and nodes {5, 6}, while the least entangled is the partition between modes {1, 4, 5} and modes {2, 3, 6}.

### Quantum secret sharing simulations

Quantum secret sharing consists of sharing information (either quantum or classical) between several players through the use of entangled quantum states. The information is first transferred to a multipartite entangled state. Each player is then given a piece of the total entangled state, and the original information can only be retrieved through a collaboration of subsets of the players. The quantum correlations increase both the protocol security as well as its retrieval fidelity as compared to what is attainable with only classical resources^38^,^39^,^40^.

Here we demonstrate a five-partite secret sharing protocol, which uses a six mode all-optical quantum graph with the structure shown in Fig. 4. This choice of cluster was proposed in ref. 41. Nodes on the edge of the pentagon (labelled 1–5) represent the players, and the central node (6) encodes the secret before its coupling to the conglomerate state. Hence, this central information carrying node is termed the dealer.

In the present case, the nodes corresponding to the players and the dealer are associated with the annihilation operators 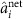, which, in turn, are constructed as a combination of the leading six squeezed eigenmodes of the comb. This transformation is obtained with the same matrix *U*_se_ that is employed to build the rightmost cluster state in Fig. 3d. The total transformation is written as:


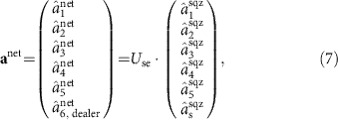


where the operators 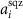 are the annihilation operators for the leading five squeezed eigenmodes of the quantum resource, as defined in previous sections. The sixth squeezed mode comprises the secret state, that is, 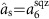.

Given this configuration, at least three players must collaborate to reconstruct the secret (see Methods section for details). Any set of three players constitutes what is termed an access party. As an example, we consider the access party of players one, two and three. To access and therefore reconstruct the 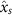 or 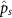 field quadrature of the secret state, the three players within this access party must each measure a specific quadrature of their local fields 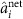, and combine their independently obtained results with the dealer's *p* quadrature measurement in the following access party operators:


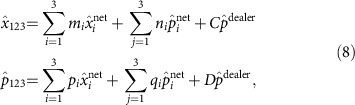


where the coefficients *m*_*i*_, *n*_*i*_, *p*_*i*_, *q*_*i*_, *C* and *D* are real. The value of these coefficients, and thus the specific linear combination between the measurements, is dictated by the condition that the final result must contain only field quadratures of the secret as well as squeezed quadratures of the input resource. Importantly, any linear combination that results in the measurement of an anti-squeezed quadrature of the input resource must be avoided. These conditions ensure that in the limit of infinite squeezing, the statistics of the measurement precisely reflect those of the secret state. After rewriting the access party quadrature measurements under these conditions, one finds the following form for the access party operators:


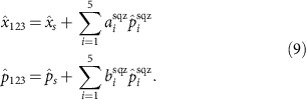


Thus, the combined measurements of the access party and the dealer yield an estimation of the secret, whose retrieval fidelity directly depends on the degree of input squeezing and the choice of the *U*_se_ matrix. More precisely, if the unitary *U*_se_ is completely general (i.e., not associated with the pentagonal cluster examined in the present case), it is not guaranteed that the access party quadrature combinations can be written in a form consisting of only squeezing quadratures of the resource state as in equation (9). For the situation in which such a form is indeed possible, the corresponding graph may be utilized for secret sharing as in the present case. It also then becomes possible to demonstrate that no solution exists for groups of only two players, which implies that two players alone can not recover the secret by virtue of the fact that the contribution of the anti-squeezing quadratures can not be fully removed, which corrupts their individual measurements even in the limit of infinite squeezing (see Methods for details).

In a genuine secret sharing scenario, 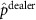 is measured first, and the result is broadcast to the players via a classical channel, thus implementing the encoding of the secret state onto the players graph. In our case, the quadratures of the secret are reconstructed by shaping the LO to coincide with the linear combination of resource modes described in equation (8). To assess the quality of the secret sharing simulation, we measured the residual noise associated with 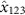 and 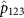 (see Methods for details). These noise variances are measured for two different multimode squeezing resources. For the first case, the quantum source is operated in a configuration that contains −6.6 dB (corrected for losses) of squeezing in the leading mode (this corresponds to the squeezers seen in Fig. 2b). In the second case, the overall squeezing is decreased by appropriately adjusting the pump power driving the parametric process, such that the leading squeezer exhibits a noise reduction of −4.5 dB (corrected for losses) relative to the vacuum level. The distribution of noise variances for the squeezers is the same in both situations and follows the trend observed in Fig. 2b. The retrieval fidelities for all 10 possible access party combinations are determined by measuring the noise variances prescribed by equation (8) and are displayed in Fig. 4. For purposes of comparison, the same access party noise variances are also measured in the absence of squeezing (that is, the resource state is a vacuum state), which are also shown in Fig. 4. As expected, the mean value for these retrieval fidelities is ∼0.60, which corresponds to the classical limit^42^,^43^ (more details in the Methods section). With quantum resources, however, we observe fidelities higher than the classical limit, which increase with better squeezing.

The accuracy with which the pulse shaper sculpts the field combinations dictated by equation (8) is also assessed by directly calculating the expected fidelities based on the known input squeezing levels with the help of equation (9). These calculated fidelities are displayed as the black curves in Fig. 4 for each of the two utilized multimode resources. The agreement between these calculated fidelities and the experimentally-measured ones is generally good. The origin of deviations between the two curves arises from the fact that spectrally dependent losses encountered in the production and detection of the multimode state do not allow the amplitude and phase quadratures of the covariance matrix to be simultaneously diagonalized^18^. As a result, the spectral form of the eigenmodes for the two quadratures is slightly different, and this corrupts the perfect cancellation of the anti-squeezing contribution in equation (4). This effect is more present with a higher level of squeezing, as the influence of losses becomes more significant. In principle, these deviations may be reduced by minimizing spectrally dependent losses in the generation and detection of the quantum source. Nonetheless, the general agreement between the experimentally measured and calculated variances confirms the utility of the apparatus at simulating arbitrary mode constructions. Despite the fact that the input secret can not be varied, as is usual in demonstrations of quantum secret sharing, this study allows for implementing secret sharing protocols consisting of a large number of modes while also exploring the influence of parameters such as loss and squeezing values.

## Discussion

In summary, we have experimentally implemented a versatile and scalable detection scheme that allows for on-demand simulation of realisation of all-optical quantum graphs. This approach permits a direct interrogation of all of the relevant information that characterizes a multimode Gaussian state in a user-defined basis. Examples of such a synthesis include cluster state generation as well as a multipartite quantum secret sharing protocol that is built on a six-node cluster graph.

Importantly, the creation of these cluster graph states with our system does not necessitate any change in the optical architecture. Rather, the connectivity of the structure is varied by simply modifying the basis in which the state is detected. Given that an arbitrary, multimode Gaussian transformation of a set of squeezers can be achieved with a unitary matrix, a set of identifiable pulse shapes may be associated with the transformation output. In this manner, it is possible to directly probe any Gaussian entanglement criteria. The fact that each of these structures is revealed by only adjusting the measurement basis indicates that these graphs are all implicitly embedded within the multimode entangled resource. Furthermore, this approach allows for the implementation of any quadratic hamiltonian, modulo the available ressources which are the squeezing eigenvalues. In that sense, our system consists in a first step towards a quantum simulator as it allows for probing any multimode system with quadratic evolution.

On the other hand, the current implementation is not yet compatible with measurement based quantum computing, as only one mode can be measured at a time. However, multimode homodyne detection can be directly implemented, transferring what has been achieved in the spatial domain^15^ to the frequency domain. In conjunction with post processing this has been demonstrated to be a versatile universal Gaussian MBQC system^16^,^34^. Finally, any quantum computing application demonstrating quantum supremacy requires going beyond the gaussian statistics, which can be efficiently simulated with a classical computer. In our system, non-gaussian operation can be readily implemented using the so called Quantum Pulse Gate^44^ which allows for mode-dependent photon subtraction^45^. This would turn our system into a unique highly versatile multimode non-gaussian source compatible with MBQC applications.

## Methods

### Quadrature operator definition

The amplitude and phase quadrature operators are defined, respectively, by 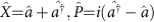. Thus, the variances of the amplitude and phase quadrature operators for a vacuum state are equal to one in our work.

### Detection and data acquisition

Light detection is achieved with balanced homodyne detection, which is performed with selected silicon photodiodes that exhibit ∼99% quantum efficiency and a bandwidth of ∼100 MHz. The homodyne fringe visibility is ∼93–95%, and the total loss for the detection of squeezing is ∼15%. The photocurrent difference is amplified with a commercial amplifier (model Mini-Circuits ZFL-500LN) and then demodulated at 1 MHz. Each squeezing curve is measured following ∼1 s of data acquisition. Hence, an *n*-dimensional covariance matrix is fully measured in *n*·(*n*+1)/2 s or ∼2 min for the 16-dimensional matrix shown in the present work.

### Optimization of unitary cluster matrix

For cluster states, one can demonstrate that if *U*_net_ in equation (4) is a unitary matrix that leads to a cluster defined by its adjacency matrix^13^, then the application of an arbitrary orthogonal matrix 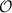 to the unitary matrix (that is, 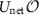) also leads to the same graph cluster state^34^. Due to the non-uniform squeezing distribution of our multimode quantum resource (as seen in Fig. 2), the measured nullifier variances are dependent on the specific choice of the unitary transformation. To equally distribute the finite squeezing resources amongst the targeted cluster, an evolutionary algorithm is utilized to search for the matrix 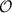 that minimizes the mean nullifier variance based on the measured covariance matrix.

### Quantum secret sharing protocol

For the secret sharing protocol presented in Fig. 4, the corresponding six-node cluster matrix *U*_se_ used in equation (7) has real part *X*_se_


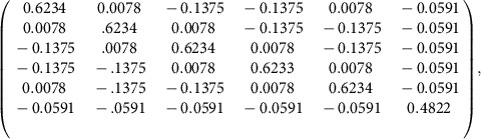


and the corresponding imaginary part, *Y*_se_, is


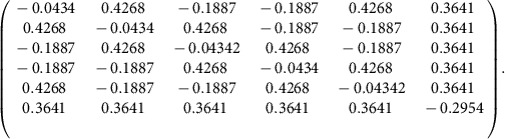


Its action on the quadrature operator is represented by the symplectic matrix


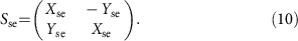


The graph quadrature operators are then obtained as


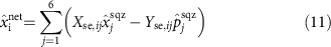



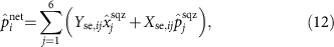


which are actually a set of twelve equations expressing the local quadratures given to the players (*i*=1, ..., 5) and the dealer (*i*=6). The secret is encoded in the sixth squeezed mode. To explain how the secret quadratures are measured by an access party, let us concentrate on a specific one, namely the one composed by players one, two and three as in the main text. Players are allowed to measure either the local position or momentum quadrature, or a rotated version of the two. They may then collaborate, linearly combining their outcomes. Moreover, the dealer measures 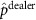 and broadcasts the result to all the players. In practice, our experiment measured the local quadratures of each access party and the dealer's momentum quadrature at the same time by a suitable shaping of the local oscillator; nonetheless, we will detail the procedure to retrieve the secret quadrature in the scenario outlined above. Importantly, the result does not change.

Let us consider the access party of players one, two and three. Assume that the dealer measures 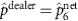 getting the result *μ*. As a consequence, the last terms of equations (11) and (12) dictate a relation between the initially squeezed quadratures and the secret quadratures. We can use this new relation to rewrite one of the anti-squeezed quadratures, say 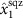 in terms of *μ*, the five remaining anti-squeezed quadratures 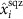, and all six of the squeezed quadratures 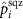. The first three components of both equations (11) and (12) are rewrritten as (*i*=1, 2, 3)


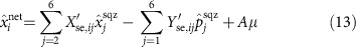



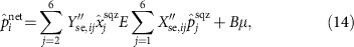


where *A* and *B* are real numbers. To reconstruct one of the secret quadratures, say 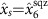, the players need to consider linear combinations of the local operators 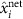 and 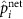 of the form


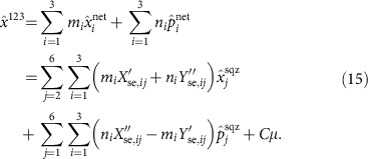


*C* is a real number which depends on the coefficients *m*_*i*_ and *n*_*i*_. The goal of the players is to find coefficients *m*_*i*_ and *n*_*i*_ such that


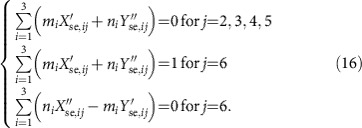


As such, 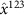 will not contain the anti-squeezed quadratures, and the coefficient of the secret momentum quadrature 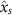 is one. If a solution of the linear system (16) exists, the access party has access to the measurement of


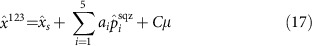


where the *a*_*i*_'s are fixed by the solution of (16). The real number *C*_*μ*_ is known since *μ* is broadcasted by the dealer. Thus, with classical post-processing, the access party can measure


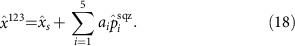


A similar reasoning allows the access party to measure 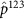 as in the main text. We checked numerically that a solution exists for both 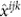 and 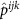 for every possible access party. Also, we verified that no solution exists when any pair of players is considered. Consequently, no less than three players can avoid the anti-squeezed quadratures, which spoils a retrieval of the secret quadrature.

To assess the quality of a secret sharing protocol carried out with our resource, we compute the fidelity between a general input coherent state and the state reconstructed from many measurements of the secret quadratures. We make use of the following formula for the fidelity between two Gaussian states^46^


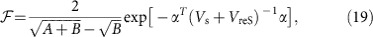


where *V*_s_ and *V*_reS_ are the covariance matrices of the input secret and reconstructed secret, respectively; *A*=det(*V*_s_+*V*_reS_), *B*=(det*V*_s_−1)(det*V*_reS_−1); and *α* is the difference of the mean amplitudes of the two Gaussian states. When the secret is squeezed vacuum, or when the mean field can be retrieved exactly, *α*=0, which permits the fidelity to be recast as


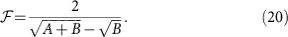


The covariance matrix of the reconstructed secret state and of the initial secret are


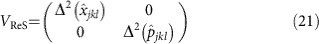


and


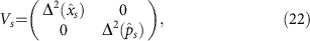


respectively, where *VreS* is measured according to equation (9) and (*jkl*) is any access party. From equation (9), since the modes are independently squeezed at the beginning, the variances of the reconstructed quadratures are computed as


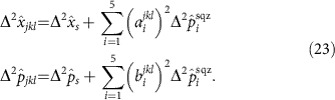


**Figure 5:**
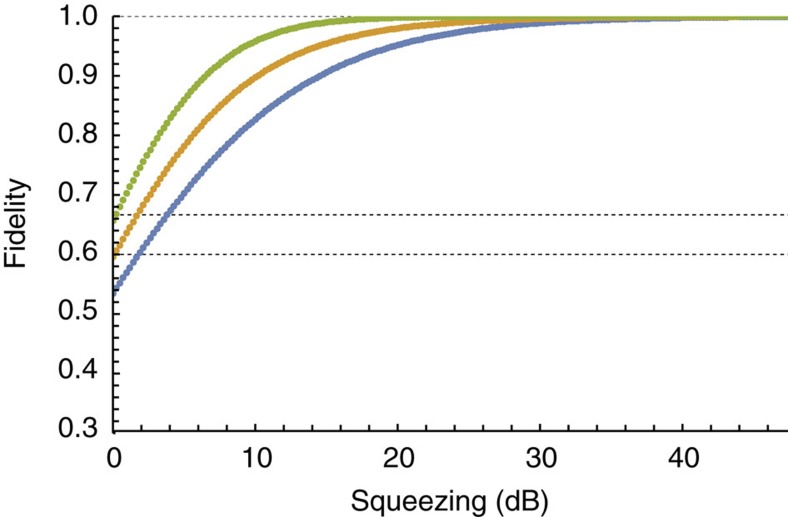
Theoretical fidelity between the secret and the reconstructed state. In a three access party/five player secret sharing protocol, the data were obtained assuming the ratio between the squeezing parameters of the modes used to build the graph is fixed, and the overall squeezing level is controlled with a common scaling factor. The horizontal axis is the squeezing level of the most squeezed mode. The top line (green) is the highest fidelity among all the possible access parties while the bottom line (blue) represents the worst. The line in the middle (orange) was obtained by averaging the fidelity over all access parties.

Figure 5 is obtained from equation (23) under the assumption that the secret is a coherent state and the squeezing ratio between the modes underlying the graph is fixed and follows the distribution seen in Fig. 2. The overall squeezing is thus adjusted with a common scaling factor. If no squeezing is present in the resource, the best retrieval fidelity among the access parties approaches 2/3, which is consistent with the teleportation limit achievable with classical resources^42^. Likewise, the average fidelity approaches 3/5, consistent with the *k*/*n* classical limit for threshold schemes of quantum secret sharing^43^. Both the maximum and the average fidelity, as well as the minimum fidelity across the access parties, approach a value of unity as the overall squeezing level increases.

To obtain the black dot-dashed curves in Fig. 4, we drew Gaussian-distributed random values with s.d.'s matching those of the experimentally measured quadrature squeezing values. Using these random numbers, numerical fidelities are obtained by simulating the secret sharing process with the use of equation (20).

### Data availability

The data that support the findings of this study are available from the corresponding author on request.

## Additional information

**How to cite this article:** Cai, Y. *et al*. Multimode entanglement in reconfigurable graph states using optical frequency combs. *Nat. Commun.* **8**, 15645 doi:10.1038/ncomms15645 (2017).

**Publisher's note**: Springer Nature remains neutral with regard to jurisdictional claims in published maps and institutional affiliations.
